# MALDI-IHC-Guided In-Depth Spatial Proteomics: Targeted and Untargeted MSI Combined

**DOI:** 10.1021/acs.analchem.2c04220

**Published:** 2023-01-13

**Authors:** Britt S. R. Claes, Kasper K. Krestensen, Gargey Yagnik, Andrej Grgic, Christel Kuik, Mark J. Lim, Kenneth J. Rothschild, Michiel Vandenbosch, Ron M. A. Heeren

**Affiliations:** † The Maastricht MultiModal Molecular Imaging (M4I) institute, Division of Imaging Mass Spectrometry (IMS), Maastricht University, 6229 ER Maastricht, The Netherlands; ‡ AmberGen, Inc., 44 Manning Road, Billerica, Massachusetts 01821, United States; § Molecular Biophysics Laboratory, Department of Physics and Photonics Center, Boston University, Boston, Massachusetts 02215, United States

## Abstract

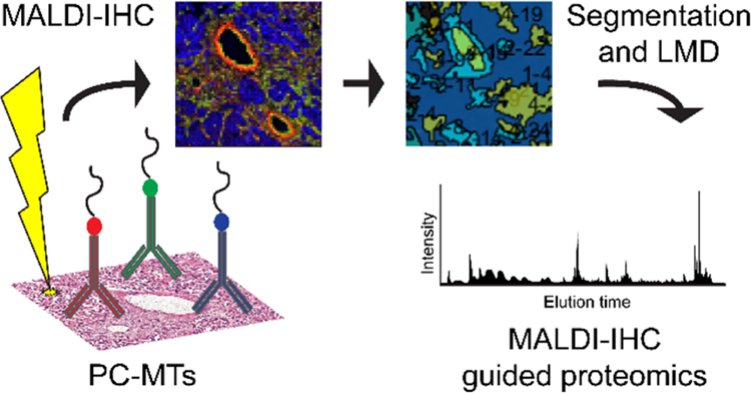

Recently, a novel technology was published, utilizing the strengths of matrix-assisted laser desorption/ionization (MALDI) mass spectrometry imaging (MSI) and immunohistochemistry (IHC), achieving highly multiplexed, targeted imaging of biomolecules in tissue. This new technique, called MALDI-IHC, opened up workflows to target molecules of interest using MALDI-MSI that are usually targeted by standard IHC. In this paper, the utility of targeted MALDI-IHC and its complementarity with untargeted on-tissue bottom-up spatial proteomics is explored using breast cancer tissue. Furthermore, the MALDI-2 effect was investigated and demonstrated to improve MALDI-IHC. Formalin-fixed paraffin-embedded (FFPE) human breast cancer tissue sections were stained for multiplex MALDI-IHC with six photocleavable mass-tagged (PC-MT) antibodies constituting a breast cancer antibody panel (CD20, actin-αSM, HER2, CD68, vimentin, and panCK). K-means spatial clusters were created based on the MALDI-IHC images and cut out using laser-capture microdissection (LMD) for further untargeted LC-MS-based bottom-up proteomics analyses. Numerous peptides could be tentatively assigned to multiple proteins, of which three proteins were also part of the antibody panel (vimentin, keratins, and actin). Post-ionization with MALDI-2 showed an increased intensity of the PC-MTs and suggests options for the development of new mass-tags. Although the on-tissue digestion covered a wider range of proteins, the MALDI-IHC allowed for easy and straightforward identification of proteins that were not detected in untargeted approaches. The combination of the multiplexed MALDI-IHC with image-guided proteomics showed great potential to further investigate diseases by providing complementary information from the same tissue section and without the need for customized instrumentation.

## Introduction

Immunohistochemistry (IHC) is one of the most commonly used imaging modalities to obtain the spatial distribution of biomolecules in the context of the native tissue. Combined with fluorescence microscopy, it is possible to obtain a spatial resolution that allows subcellular localization of a biomarker of interest. With fluorescent multiplex IHC methods, it is even possible to visualize between three to eight biological markers on the same tissue providing valuable information on the molecular organization for diagnostics, research, and more.^1,2^ While imaging multiple markers is possible using IHC, the upper limit of targets is quickly reached, as the fluorophores would overlap in their respective excitation and emission bands.^3^ This results in spectral overlap and cross-reactivity, as well as reduces the specificity when multiple fluorophores are used simultaneously. Additionally, traditional multiplexing methods require iterative workflows where numerous probes are added and removed repeatedly (PerkinElmer’s OPAL multispectral platform, *t*-CyCIF,^4^ and CODEX^5^). These types of workflows are very time-consuming and due to the iterative nature of the workflow, the risk of confounding results from incomplete or unsuccessful cycles has to be considered. Additional multiplexing methods to consider are imaging mass cytometry (IMC) and multiple ion beam imaging (MIBI). These modalities overcome the issue of overlapping fluorophores and iterative workflows by measuring the abundance of monoisotopic metal isotopes linked to antibodies using a time-of-flight inductively coupled plasma mass spectrometry (CyTOF)^6^ or secondary-ion mass spectrometry (SIMS).^7^ While IMC and MIBI allow multiplexing of up to 40 targets at subcellular resolution,^8,9^ it is very time-consuming (around 20 Hz for stable measurements) and in the case of IMC destructive to the tissue, thus not allowing for follow-up analysis.

Alternatively, matrix-assisted laser desorption/ionization (MALDI) mass spectrometry imaging (MSI) allows almost unlimited multiplexing through its unlabeled molecular imaging capabilities.^10^ In brief, a tissue section is scanned in a gridlike fashion with a laser, ablating and ionizing the molecules in the tissue. The resulting ions are sent to a mass spectrometer, creating a single mass spectrum of the molecular composition for each of the pixels within the tissue grid, which results in a spatial image of all detected masses within the tissue. Thus, MALDI-MSI provides spatial information of biomolecules, such as lipids and metabolites, in their original tissue environment. This information can be valuable on its own or can be used to determine regions of interest (ROIs) for further investigation with an in-depth spatial omics workflow. Focusing on proteins, this further characterization can be done with either top-down^11^ or bottom-up as described in this manuscript. The bottom-up approach uses a laser-capture microdissection (LMD) microscope to cut out and collect small areas of the MALDI-measured tissue for LC-MS proteomics analysis. Recently, spatial omics has grown in popularity as it provides an unparalleled set of data, combining molecular spatial information with proteomics data.^12,13^ MALDI-MSI can also directly reveal untargeted spatial information of proteins when combined with untargeted on-tissue digestion.^14^ This allows for imaging of peptides in a bottom-up fashion, providing a broad overview of the spatial proteomics profile in tissue, however lacking the specificity offered by classic IHC.^15^

With commercial instruments, a spatial resolution of 5–10 μm can be achieved routinely. However, as the pixel size decreases with the spatial resolution, the amount of ablated material decreases quadratically with it, resulting in limited ion yields, and combined with ion suppression effects, it limits the sensitivity of the methods.^16^ Post-ionization with MALDI-2 is a recent development within MALDI-MSI, which increases the ionization of certain biomolecules in the ablated ion plume, thus circumventing issues of low ion yield and allowing for more broadly applied high-resolution measurements.^17^

In a newly developed workflow, dubbed matrix-assisted laser desorption/ionization immunohistochemistry (MALDI-IHC), photocleavable mass-tags (PC-MTs) conjugated to antibodies or lectins are used to target molecules of interest, similar to standard IHC using fluorescently labeled antibodies.^18^ When the PC-MT-antibody probes are subsequently subjected to UV light, which is performed prior to matrix deposition and MSI, the linker is efficiently cleaved, resulting in the release of the ionizable peptide mass reporter and thus allowing targeted imaging of the selected markers with MALDI-MSI. Thus, MALDI-IHC utilizes the strengths of MALDI-MSI to achieve highly multiplexed, targeted imaging of biomolecules in tissue.^18^ Earlier MSI work also utilized photocleavable mass-tag conjugated probes.^19^ However, the design of the photocleavable mass-tags resulted in the bulk of the photocleavable linker remaining on the mass reporter after photocleavage. In addition, labeling of the probe with the photocleavable mass-tag is a multistep process. In contrast, the design of the MALDI-IHC photocleavable mass-tags simplifies conjugation and increases the sensitivity, multiplex capabilities, and overall utility of this approach.^18^ Compared with already existing multiplexing methods, MALDI-IHC has the advantage that no cyclic workflows are required, and it is nondestructive, making it compatible with subsequent measurements, e.g., untargeted peptide MALDI-MSI of the same tissue section or spatially targeted proteomics through spatial omics. Additionally, the measurement time is quick (up to 10 kHz, depending on the mass spectrometer used), requires no specialized MS instrumentation and antibody labeling is an easy process.^18^

Herein, we demonstrate the utility of MALDI-IHC and explore the complementarity of untargeted on-tissue bottom-up spatial proteomics and targeted MALDI-IHC for studying specific markers of the tumor microenvironment of breast cancer tissue. Furthermore, we exploited the effect of MALDI-2 on the PC-MT-antibodies and expanded the MALDI-MSI to a multiomics workflow using MALDI-IHC-guided proteomics.

## Methods

### Chemicals

Water (HPLC and ULC/MS grade), ethanol, acetonitrile (ACN), trifluoroacetic acid (TFA), and xylene were obtained from Biosolve B.V. (Valkenswaard, The Netherlands). α-Cyano-4-hydroxycinnamic acid (CHCA), citraconic anhydride, potassium sulfate, 2,5-dihydroxybenzoic acid (DHB) 98%, and formic acid (FA) >95% were obtained from Sigma-Aldrich (St. Louis, MIA). Hydrochloric acid fuming 37% was obtained from Supelco (Bellefonte, PA). Trypsin was obtained from Promega (Sequencing Grade Modified Trypsin, Madison, WI). Hematoxylin and Entellan were obtained from Merck (Darmstadt, Germany). Eosin Y was obtained from J.T. Baker (Center Valley, PA).

### Sample Preparation

Human breast cancer formalin-fixed paraffin-embedded (FFPE) tissue blocks from two separate patients, patient 1 and patient 2 (OriGene Technologies, Inc.; Table S1) were sectioned at 3 μm thickness and mounted on Intellislides (Bruker Daltonics GmbH, Bremen, Germany). For the multiplex MALDI-IHC, tissues were stained with six PC-MT antibody probes (Table S2) of a breast cancer antibody panel (CD20, α-smooth muscle actin (actin-αSM), human epidermal growth factor receptor 2 (HER2), CD68, vimentin, and pan-cytokeratin (panCK)) using a protocol as previously reported.^18^ The hormone receptor status includes three receptors, the estrogen receptor (ER), the progesterone receptor (PR), and the HER2 receptor, and can either be positive (+) if present or negative (−) if absent.^20^ The hormone receptor status of the patient samples used was predetermined by the supplier as: patient 1 (PR+/ER+/HER2−) and patient 2 (PR–/ER–/HER2+). Both unstained and prestained slides were shipped and stored in a desiccator at room temperature until further experiments.

For the on-tissue digestion, the untreated FFPE tissue was first deparaffinized in an oven (60 °C for 60 min) followed by a series of washing steps with xylene (2 × 5 min), in ice-cold 100% ethanol (1 × 3 min and 1 × 1 min), ice-cold 96% ethanol (1 min), ice-cold 70% ethanol (1 min), and 4 °C HPLC water (2 × 3 min). Antigen retrieval was performed using the Retriever 2100 (Aptum Biologics Ltd, Rownhams, U.K.) for 20 min at 121 °C. The citraconic anhydride buffer (pH = 3) was prepared as described by Drake et al.^21^ The trypsin solution was freshly prepared by adding 200 μL of the cold HPLC-grade water to 20 μg of trypsin. The enzyme was sprayed with the HTX M3+ sprayer (HTX Technologies LLC, Carrboro). Spraying parameters were as followed: temperature = 45 °C, nozzle velocity = 1200 mm/min, flow rate = 30 μL/min, trypsin concentration = 0.1 μg/μL, number of passes = 8, track spacing = 2.5 mm, and nitrogen gas pressure of 10 psi. The slide was then put in an incubation chamber at 37.5 °C for 16 h. CHCA matrix solution (10 mg/mL in 70% ACN + 1% TFA) was applied with the HTX M3+ sprayer (HTX Technologies LLC, Carrboro) using the following settings: temperature = 75 °C, nozzle velocity = 1200 mm/min, flow rate = 120 μL/min, CHCA concentration = 10 μg/μL, number of passes = 4, track spacing = 1.5 mm, and nitrogen gas pressure of 10 psi.

The PC-MT antibody probes on the prestained slides were photocleaved by illumination of UV light at 365 nm with a Phrozen UV curing lamp for 10 min (3 mW/cm^2^) to achieve maximum photocleavage based on AmberGen’s optimized protocol and on an earlier study,^22^ followed by matrix sublimation of 50 mg DHB at 160 °C for 180 s (HTX Sublimator, HTX Technologies, Chapel Hill, NC). The matrix was recrystallized in an oven (50 °C, 90 s) using a preheated Petri dish with 0.5% ethanol in water.

### MALDI-TOF Imaging

MALDI-MSI data of the digested dataset were acquired on a timsTOF fleX in positive polarity (Bruker Daltonik GmbH, Germany) at a pixel size of 20 × 20 μm, using 500 shots and a laser frequency of 10 kHz. MALDI-IHC data was acquired at the same pixel size and polarity, but using 200 shots and a laser frequency of 5000 Hz for MALDI-1 and 100 shots and 1000 Hz for MALDI-2. MALDI-IHC data were also acquired on a rapifleX MALDI Tissuetyper instrument operating in positive polarity using reflectron mode (Bruker Daltonik GmbH, Germany). Here, imaging was performed at 10 × 10 μm pixel size, using 100 shots and a laser frequency of 5000 Hz, and at 5 × 5 μm using 20 shots and a laser frequency of 500 Hz. Both instruments are equipped with a Nd:YAG laser emitting at 355 nm with a laser diameter of 5 μm. On the timsTOF fleX, the post-ionization effect was obtained by a diode-pumped solid-state NL 204-1k-FH laser (EKSPLA, Vilnius, Lithuania; wavelength: 266 nm). The distance of the PI laser beam to the sample surface was set to ∼250 μm and the delay between the two lasers’ pulses, both operated at 1 kHz, was set to 10 μs. Calibration was performed using red phosphorus prior to the imaging experiments.

### Laser-Capture Microdissection

Prior to LMD, the matrix was removed by submerging in 70% ethanol for 5 min. LMD was performed using a Leica LMD 7000 (Leica Microsystems, Wetzlar, Germany). External coordinate information of areas in the form of an XML file, based on the K-means clusters exported from SCiLS lab, were dissected using the following laser settings: power 44, aperture 15, speed 10, specimen balance 15, line spacing for draw + scan 10, head current 100%, pulse frequency 119. For the samples on IntelliSlides, the laser setting “draw and scan” was used. The dissected tissue was collected in the cap of 0.5 mL centrifuge tubes, prefilled with 20 μL of 50 mM citric acid, and immediately transferred for further analysis.

### Proteomics Sample Preparation

For each ROI removed, a total of 0.5 mm^2^ was collected for bottom-up proteomics. In brief, collected samples were centrifuged at 15,000*g* to collect everything at the bottom of the tube. Next, samples were sonicated for 10 min at room temperature (RT) and incubated at 99 °C, shaking at 800 rpm for 1 h followed by the addition of 2.2 μL of 0.1% RapiGest and incubation for 10 min at RT, shaking at 800 rpm before the addition of 2 μL of 500 mM ammonium bicarbonate (ABC). Next, samples underwent reduction and alkylation. First by the addition of 1.3 μL of DTT (200 mM in 50 mM ABC for final [DTT] = 10 mM) and incubation for 40 min at 800 rpm and 56 °C. Second, 1.4 μL of IAM (400 mM in 50 mM ABC for final [IAM] = 20 mM) was added and the sample was incubated for 10 min at 800 rpm and RT. Finally, 1.4 μL of DTT (200 mM in 50 mM ABC for final [DTT] = 10 mM) was added and the sample was incubated for 10 min at 800 rpm and RT. For digestion of proteins, 1 μL of trypsin (0.5 μg/μL for final v/v = 15 μg/mL) was added and the sample was incubated overnight for 16 h, shaking at 800 rpm at 37 °C. After the first incubation, 0.3 μL of trypsin (0.5 μg/μL for final v/v = 5 μg/mL) and 115 μL of ACN (final [ACN] = 80%) were added and the sample was incubated for 3 h, shaking at 800 rpm at 37 °C. After the second incubation, 6 μL of TFA (10%, final [TFA] = 0.5%) was added and the sample was incubated for 45 min, shaking at 800 rpm at 37 °C. Finally, the sample was centrifuged at 15,000*g* for 15 min at 4 °C and the resulting supernatant was collected and concentrated in a SpeedVac until storing at −20 °C before LC-MS analysis.

### LC-MS/MS Analysis

The LMD samples were injected into a nanoElute ultrahigh pressure LC system (Bruker Daltonik GmbH, Germany) onto a 150 mm column of ID 75 μm (Bruker FITEEN, Bruker Daltonik GmbH, Germany) packed with ReproSil 1.9 μm C18 beads, pore diameter 120 Å. The mobile phase consisted of 0.1% FA in UPLC-grade water (eluent A) and 0.1% FA in ACN (eluent B). Chromatographic separation of the peptides was achieved using a 120 min gradient at a flow rate of 400 nL/min at an oven temperature of 50 °C. First, a stepwise gradient of 2–17% eluent B was applied for 60 min, followed by an increase of 17–25% eluent B from 60 to 90 min, 25–37% eluent B from 90 to 100 min, 37–95% eluent B from 100 to 110 min, and finished by a washing step at 95% eluent B for 10 min. The CaptiveSpray nano-electrospray ion source (Bruker Daltonik GmbH, Germany) was used as an MS inlet source, operating at 180 °C with 3.0 L/min dry gas and 1.6 kV capillary voltage. Data were acquired on a timsTOF fleX instrument operated in PASEF mode. The TIMS accumulation time, as well as ramp time, was fixed at 100 ms. The ion mobility range values were 0.6–1.6 Vs/cm^2^ (1/K0), and a mass range of *m*/*z* 100–1700 was covered. Data-dependent acquisition was performed by acquiring one MS scan followed by ten subsequent PASEF MS/MS scans, each 100 ms. Furthermore, an active exclusion of 0.4 min was applied to the precursors.

### Histological Staining

Hematoxylin and eosin (H&E) staining was performed on the same sections used for MALDI-MSI experiments and the LMD. The residual matrix was removed by submerging in 70% ethanol (3 min). Both slides were submerged in 70% ethanol for a second time (3 min), followed by Milli-Q water (3 min). Staining was performed in hematoxylin (3 min), followed by rinsing under running tap water (3 min), eosin (30 s), running tap water (1 min), 100% ethanol (1 min), and xylene (30 s). Coverslips were mounted using Entallan mounting medium. Optical images were acquired at 20× magnification using the Aperio CS2 digital pathology slide scanner (Leica Biosystems, Wetzlar, Germany).

### Data Analysis

Bruker Compass flexImaging 5.1 and 7.0 (Bruker Daltonik GmbH, Bremen, Germany) and SCiLS lab 2022a (SCiLS GmbH, Bremen, Germany) were used to process the imaging data acquired. Data were TIC normalized and exported from SCiLS. Hotspot removal was applied to the images. Spectra were imported in mMass^23^ where peaks were picked after baseline correction to define the signal-to-noise ratios (S/N). MaxQuant (v. 2.0.3.0) was used to process the MS raw files for protein identification and label-free-quantification (LFQ). A database search was performed by matching the MS/MS spectra against in silico-derived fragments from the Swiss-Prot human database (downloaded on April 4, 2020). This database search was performed using an FDR of <0.01. Trypsin was used as proteolytic enzyme, and a maximum of two missed cleavages was allowed. Methionine oxidation and acetylation of protein N-terminal were chosen as variable modifications, and carbamidomethylation of cysteine was set as fixed modifications. The results from the database search were imported into Persus (v. 2.0.3.1), a statistical addition to the MaxQuant software for biomedical interpretation (http://www.perseus-framework.org/). The MaxQuant protein group list was filtered for “proteins only identified by site,” “reverse sequence,” and “possible contaminants.”

### Protein Identification (ID)

For the protein ID, the criteria set included a minimum of three peptide peaks to correspond to the certain protein. Additionally, all selected peaks for the certain protein had to have the same spatial distribution. Finally, mass error had to be 5 ppm or less and selected peaks had to have an A + 1 isotopic peak. Peptide peak lists of the proteins targeted with the breast cancer antibody panel were generated by performing in silico digestion in Expasy (https://web.expasy.org/peptide_mass/). Settings used for the in silico digestion were as follows: only [M + H]^+^ were included, trypsin was selected as the enzyme, up to two missed cleavages were allowed, and only peptides between 750 and 3000 Da were displayed.

## Results and Discussion

**Figure 1:**
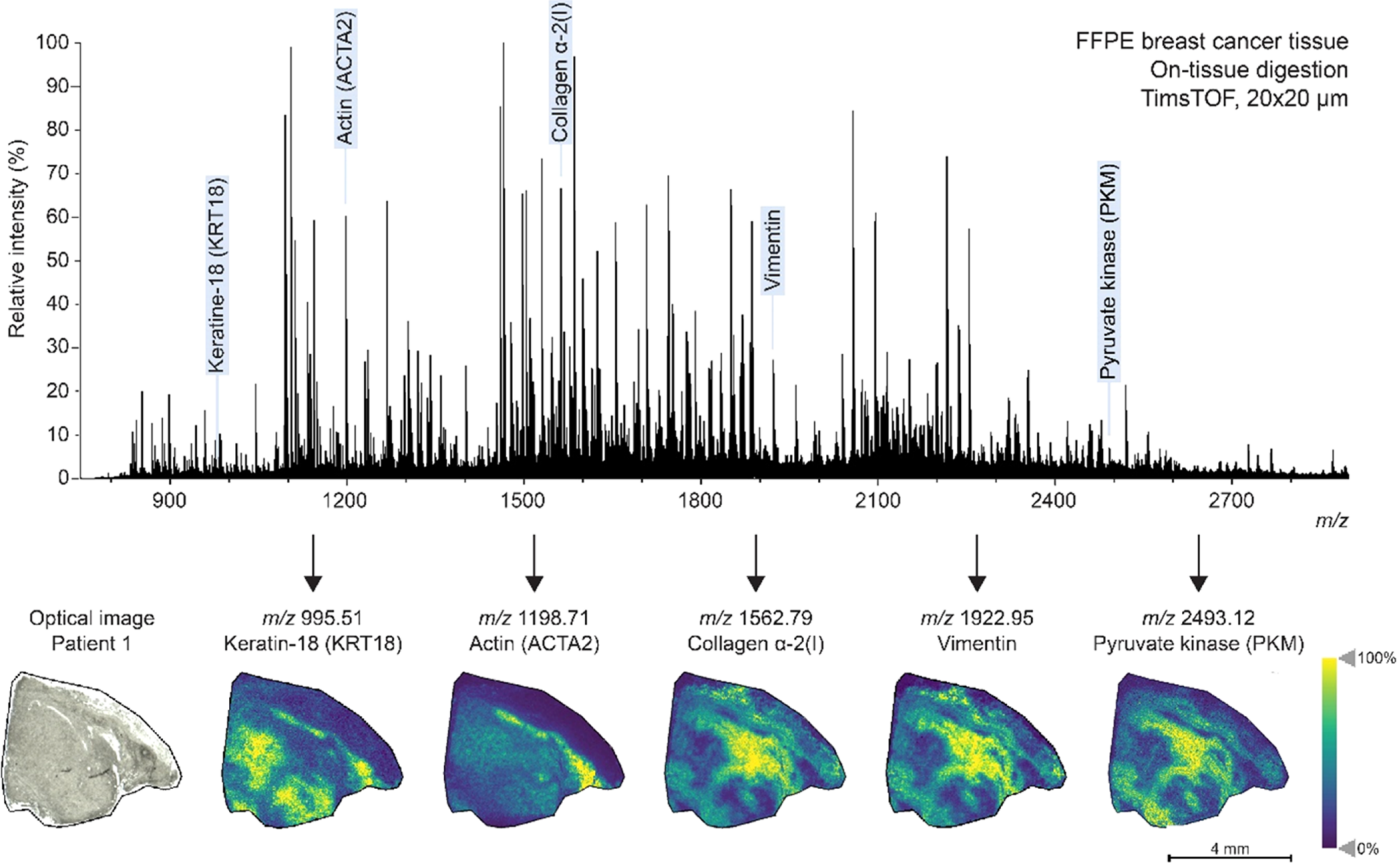
Untargeted on-tissue digestion of FFPE human breast cancer tissue (patient 1) acquired on a timsTOF. Selected masses show various distributions of multiple peptides throughout the breast cancer tissue and are highlighted with blue text. These masses were tentatively assigned based on LC-MS/MS data and in silico digestion.

Bottom-up proteomics has been a favored approach when it comes to the identification of proteins with MALDI-MSI. With the optimized trypsin digestion protocol, a spatial resolution of 20 × 20 μm was achieved on the timsTOF fleX (Figure 1). Various proteins could be identified with the LC-MS/MS showing different distributions throughout the breast cancer tissue section. With the use of in silico digestion, peptides related to the targets of the antibody panel were also investigated. This panel included the B-cell biomarker CD20,^24^ the smooth muscle cell biomarker actin-αSM^25^ for muscle and stromal cells, the breast cancer-related biomarker HER2,^20^ the biomarker CD68^26^ for macrophages, the biomarker vimentin^27^ for mesenchymal cells, and the general epithelial cell biomarker panCK.^28^ In the untargeted dataset, peptides were found related to vimentin, actin, keratin-8 (KRT8), keratin-18 (KRT18), and keratin-19 (KRT19). Additionally, as the bottom-up MALDI-MS is an untargeted approach, other proteins such as collagen-α-2 and pyruvate kinase (PKM) could be identified. Three distinct distributions were found, where collagen-α-2, vimentin, and PKM co-localized. Rothenberg et al. also showed collagen-2 and vimentin co-express in the same cells.^29^ In contrast, KRT18 showed an inverted image compared to collagen-2, vimentin, and PKM. Although panCK and vimentin are markers for different cell types, it is possible for cytokeratin-positive epithelial cells to express vimentin during epithelial–mesenchymal transition, a complex interplay known to drive metastasis.^30^ Another interesting spatial distribution was found that corresponds to fibrinogen α (FGA) and fibrinogen β (FGB) (Figure S1). Peptides related to FGA and FGB were specific for only one of the patient tissues, where the spatial distribution of both FGA and FGB obtained with MALDI-MSI localized to the necrotic area based on comparisons with the H&E of a consecutive slide.

Although some peptides could be identified, the biggest challenge for bottom-up MALDI-MSI spatial proteomics remains the identification of proteins. Even though the untargeted approach allowed for a broad range of peptides, current software solutions are inadequate when it comes to correlating the peptide peaks observed with the proteins. Additionally, general lack of standardized rules and requirements for protein ID in bottom-up MALDI-MSI proteomics can lead to big differences in reporting. Kip et al. also showed mass resolution could be a limiting factor, as FTICR data revealed almost 30% of the TOF peaks could be resolved in two or more peptide signals.^31^ Even after extensive data analysis, over 90% of the peaks in the mass spectra were left unassigned. Further development of software and databases for bottom-up MALDI-MSI proteomics could lead to a huge improvement in both number and certainty of protein ID. Additionally, it would also take the spatial distribution of peaks in consideration, the fragments observed in MS/MS spectra if available, mass accuracy, and isotopic patterns. Software such as LipostarMSI showed to be a powerful tool for combining MSI data analysis and lipid identification.^32^ However, such software is not available yet for MALDI-MSI spatial proteomics data.

### MALDI-IHC

**Figure 2:**
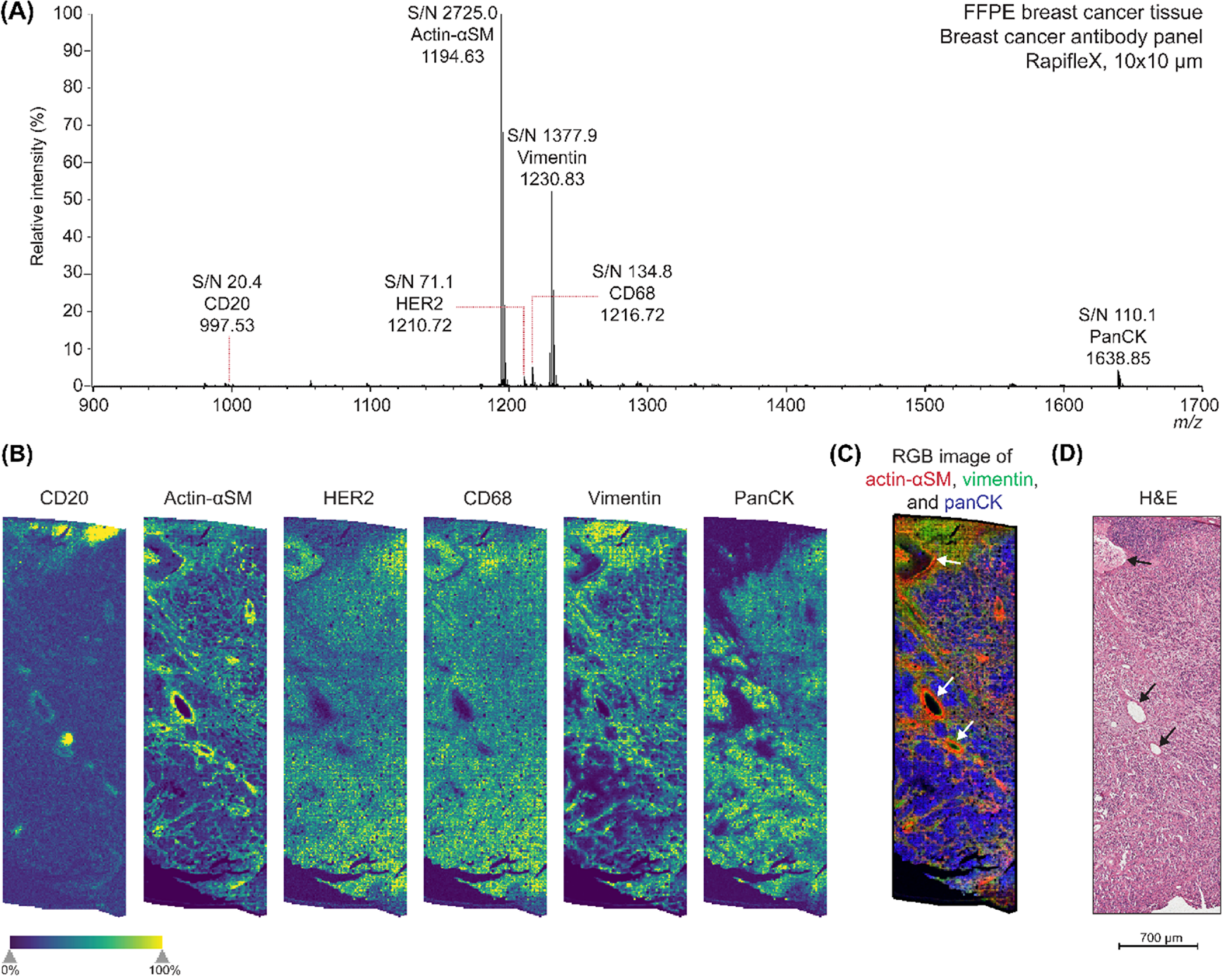
Multiplex MALDI-IHC on the rapifleX for six biomarkers in FFPE human breast cancer tissue (patient 1). (A) Average spectrum of the MALDI-IHC measurement with the breast cancer antibody panel. All six peptide mass reporters were detected. The spectrum was TIC normalized and baseline-corrected. (B) Single-ion images of the mass reporters detected, which were CD20, actin-αSM, HER2, CD68, vimentin, and panCK, respectively. (C) RGB image of actin-αSM (red), vimentin (green), and panCK (blue). (D) H&E image of a consecutive section, showing similar structures as imaged with the MALDI-IHC. White (C) and black (D) arrows point out the vascular lining of blood vessels in the tissue section, which is also highlighted by actin-αSM (B, C).

Using the PC-MTs, a six-plex MALDI-IHC was performed on consecutive FFPE human breast cancer tissue sections from patient 1. All six of the mass reporter masses were detected in the area measured (Figure 2A). A single-pixel mass spectrum at 10 × 10 μm can be found in the supplementary information (Figure S2). CD20 was detected in distinct areas of the tissue corresponding with lymph nodes or tertiary lymphoid structures (Figure 2B, panel 1). Actin-αSM and vimentin showed to have bound to the vascular lining of blood vessels and tumor stroma (Figure 2B, panels 2 and 5). In addition, vimentin showed a higher abundancy at the top area of the measurement, which corresponds to axillary lymph node tissue based on the comparison with the H&E of a consecutive section. In contrast to vimentin, panCK was negative in the axillary lymph node area and the blood vessels, but showed to be more specific to tumor cells within the tissue section (Figure 2B, panel 6). HER2 and CD68 (Figure 2B, panels 3 and 4) expression did not correspond with specific histological features in the tissue sample. This could be due to the nonspecific binding of these antibodies and therefore causing a background signal, which becomes more apparent due to the hotspot removal. According to pathology annotations provided by OriGene, this patient sample was HER2– and therefore should not show an increased abundance in regions within the tissue sample. CD68 is known to be expressed by tissue macrophages but showed a similar distribution to HER2 in this sample. As macrophages are mostly single cells distributed throughout the tissue, the spatial resolution used (10 × 10 μm) could still be too large or the signal could be below the limit of detection for imaging if macrophages were present in this sample. This could be confirmed with regular IHC, which allows for higher spatial resolution and confirmation of the specificity of the antibody bound within the cell. The RGB image of actin-αSM, vimentin, and panCK shows the different regions merged (Figure 2C) and corresponds to the different structures which were confirmed with the H&E (Figure 2D).

In contrast to the untargeted MALDI-MSI spatial proteomics, this targeted approach allowed for straightforward identification of the peaks observed due to the specific peptide mass reporters. Furthermore, since the antibodies specifically bind, this approach allows for single-cell resolution and less chance of delocalization compared to the on-tissue digestion where trypsin and matrix are applied using wet methods. The option for multiplexing shows great potential in facilitating research and allows for complementary data from the same tissue section.^18^ However, in contrast to the on-tissue digestion, these antibody panels will only be suitable for specific species and/or tissue types and are dependent on validated panels to prevent nonspecific binding of the antibodies and have a correct assay-dependent concentration.

To further investigate the single-cell resolution, a region was imaged at 5 × 5 μm spatial resolution (Figure S3). Here, the laser frequency and number of shots had to be lowered from 5000 Hz and 100 shots to 500 Hz and 20 shots to avoid oversampling that potentially results in matrix sublimation or thermal degradation at high laser fluence. Although the PC-MTs were still detected with decent signal-to-noise ratios (S/N) varying from 20.4 to 2725.0, the imaging seemed to be limited by the instrument stage as a raster pattern could be observed. New technological improvements in stage technologies, such as the microGRID (Bruker), could overcome this issue.

**Figure 3:**
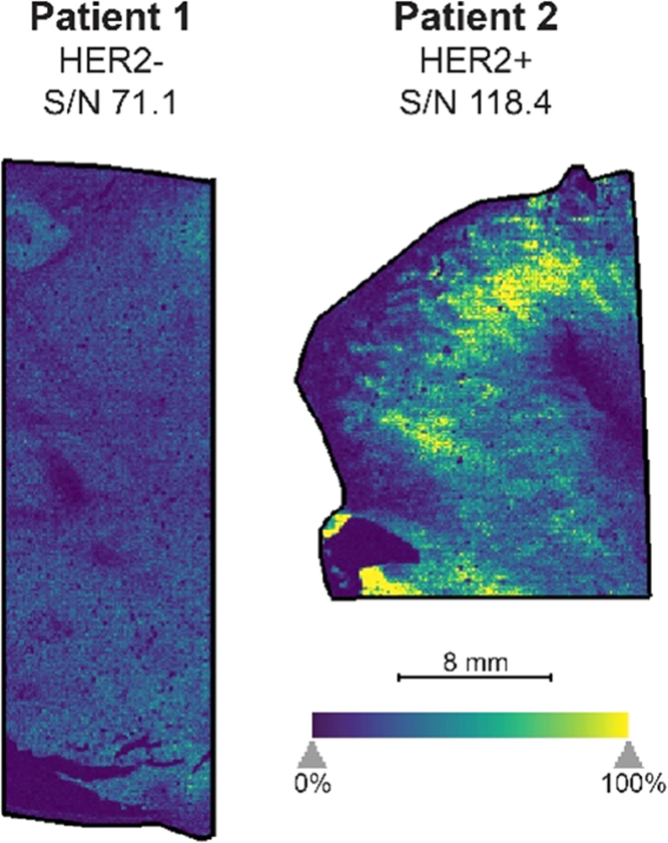
Application of MALDI-IHC to two FFPE breast cancer tissue samples to determine the HER2 status of the patients. Patient 1 (left) shows a low abundance of the HER2 mass reporter, while patient 2 (right) shows a high abundance of the HER2 mass reporter and therefore showed to be HER2+. The HER2 status (negative for patient 1, positive for patient 2) found with MALDI-IHC is consistent with the breast cancer pathology annotations provided with the samples.

Two FFPE breast cancer tissue samples with different hormone statuses were imaged to test the diagnostic applicability of MALDI-IHC. The HER2 status of these patients could be easily determined when the mass reporter for HER2 was visualized (*m*/*z* 1210.72) (Figure 3). Patient 1 showed a low abundance, while patient 2 showed a high abundance of the HER2 peptide mass reporter in the sample area measured. This is consistent with the breast cancer pathology annotations provided by OriGene, where patient 1 was reported to be PR+/ER+/HER2– and patient 2 was reported to be PR–/ER–/HER2+, and therefore patient 2 should show an increased abundance compared to patient 1 due to the presence and absence of the HER2 hormone receptor, respectively. To test the accuracy of the MALDI-IHC, it was compared to fluorescence IHC for the HER2-positive patient sample using dual-tagged Miralys antibody probes for HER2^18^ which yielded excellent agreement (Figure S4 and Table S2). This example highlights the potential of MALDI-IHC as an interesting tool to promote patient stratification or provide personalized information with a diagnostic value.

### MALDI-1 vs MALDI-2

**Figure 4:**
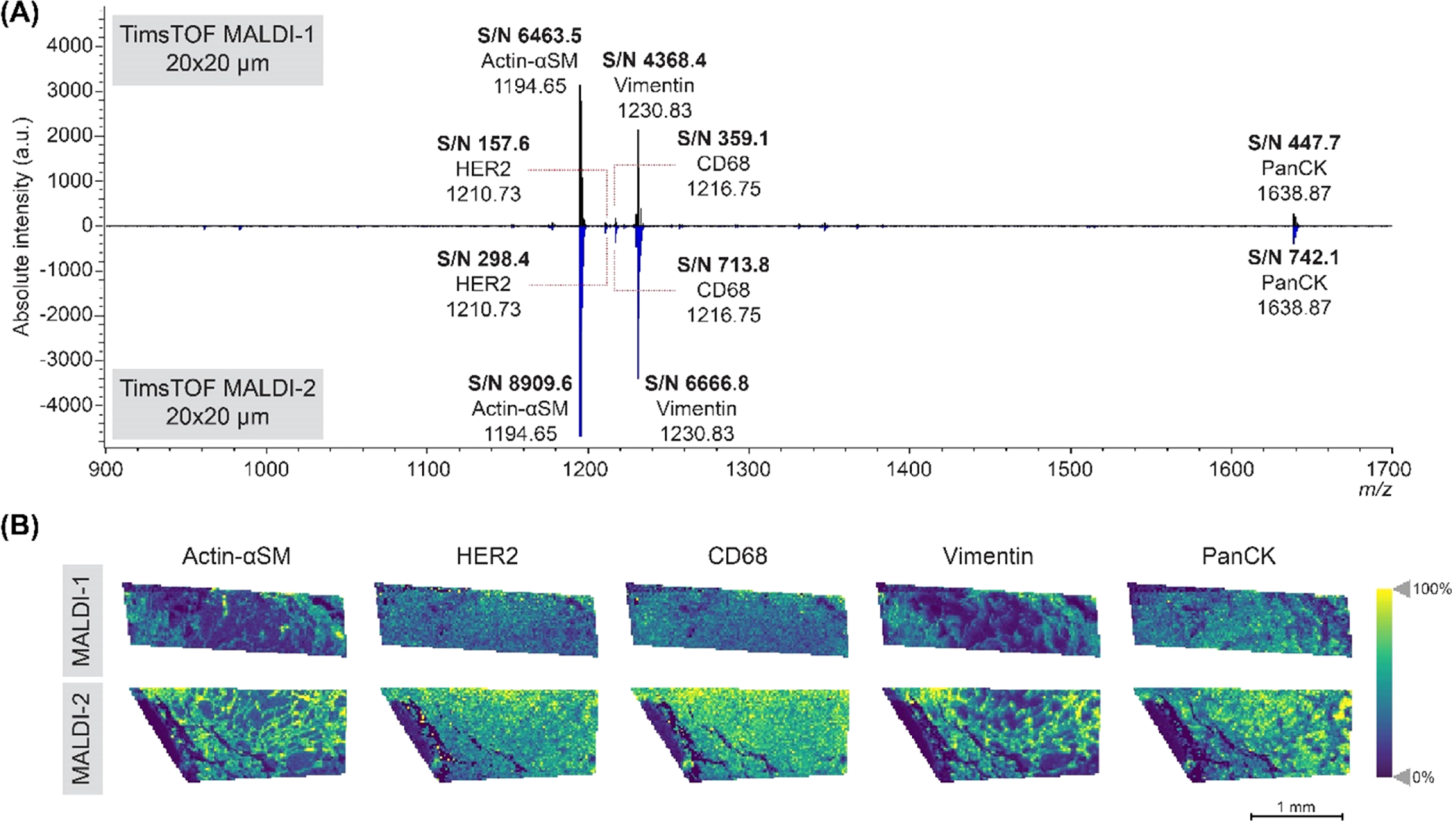
TimsTOF MALDI-1 *vs* MALDI-2 on breast cancer tissue samples from patient 1 with the PC-MTs. (A) Spectra of timsTOF MALDI-1 (top, black) and timsTOF MALDI-2 (bottom, blue) at 20 × 20 μm. The S/N of the mass-tags was increased in the MALDI-2 measurement compared to the MALDI-1 data. (B) Single-ion images of five peptide mass reporters detected, which were actin-αSM, HER2, CD68, vimentin, and panCK, respectively. The MALDI-2 data showed an increased intensity and better observable spatial details compared to the MALDI-1 data.

The effect of post-ionization with MALDI-2 was evaluated on the PC-MTs on the breast cancer sample from patient 1. MALDI-2 is known to improve the ionization of certain molecules and found to improve the intensity and S/N of the peptide mass reporters detected by a factor of two for some PC-MTs (Figure 4A). The increased intensity of the mass reporters improved the image quality and contrast of the distributions (Figure 4B). It must be noted that there could also be a biological variation as a different, although adjacent, region was measured on the tissue section. Further research on the MALDI-2 effect is needed, but this showed to be an interesting area for the development of new mass-tags.

### MALDI-IHC Guided Proteomics

Based on the MALDI-IHC images, K-means clusters were created in SCiLS Lab software to computationally create clusters of spectral similarity, which can be indistinguishable by histology alone. For each pixel in the image, the spectrum is analyzed, and based on the intensity of different peaks, the pixel is then allocated to a specific cluster, which in turn can be interpreted to represent molecular similarity.^33^ For each sample, bisecting K-means clusters could be created to correlate with distinct MALDI-IHC markers, and based on these clusters, regions were cut out of the tissue and collected for local area proteomics analysis. Regions based on rapifleX measurements were cut out directly based on the cluster shape, while regions from timsTOF could not be directly correlated due to software limitations and therefore cut out in circular shapes (Figure S5).

With LC-MS, a total of 627 unique proteins were identified across all samples after filtering common contaminants and low-confidence hits. Three of the six MALDI-IHC target proteins (actin-αSM, vimentin, and panCK-related proteins) were also identified but were not exclusive to their respective cluster region, which was expected based on their ubiquitous localization in the MALDI-IHC images. Interestingly, an inverse correlation was observed between measured protein abundance with untargeted LC-MS and targeted abundance based on MALDI-IHC peptide marker intensity. Regions cut out based on the clusters correlating mainly with panCK, vimentin, and actin-αSM localization, respectively, showed the lowest LFQ intensity for each respective marker, contradicting the abundance of the PC-MTs based on the imaging. This could indicate that binding of the probes to the target protein blocks the interaction with trypsin, thus resulting in a reduction of ionizable tryptic peptides for the target proteins. This is unexpected, as antibody binding is a reversible action and a general loss in affinity is observed at high temperatures. However, since our samples were rapidly brought to 99 °C for 1 h, the observed results can possibly be explained by denaturation of the antibody and aggregate formation with the target protein, again hindering cleavage by trypsin. This potential mechanism of trypsin blocking and aggregate formation has previously been investigated, but further experiments are necessary to confirm this.^34,35^ Furthermore, it would be interesting to explore alternative ways of breaking the antibody–antigen complexes, such as changing pH or inducing temperature fluctuations.^36^

To investigate the compatibility of the two bottom-up proteomics approaches used here, the overlapping peaks between the MALDI-IHC guided LMD followed by LC-MS/MS analysis and direct on-tissue trypsin digestion followed by MALDI imaging were compared. In total, after analysis, the MALDI-IHC-guided LC-MS/MS results identified a total of 4341 peptides, almost 10× the amount of peaks detected in the on-tissue digested MALDI spectrum after peak picking. Although a different number of peaks were detected with each approach, there was an overlap of 211 peptide masses between the two techniques (Figure S6). The big difference in identified peaks highlights the current challenge for identification with MALDI on-tissue digestion and why LC-MS remains the gold standard for protein identification.

**Figure 5:**
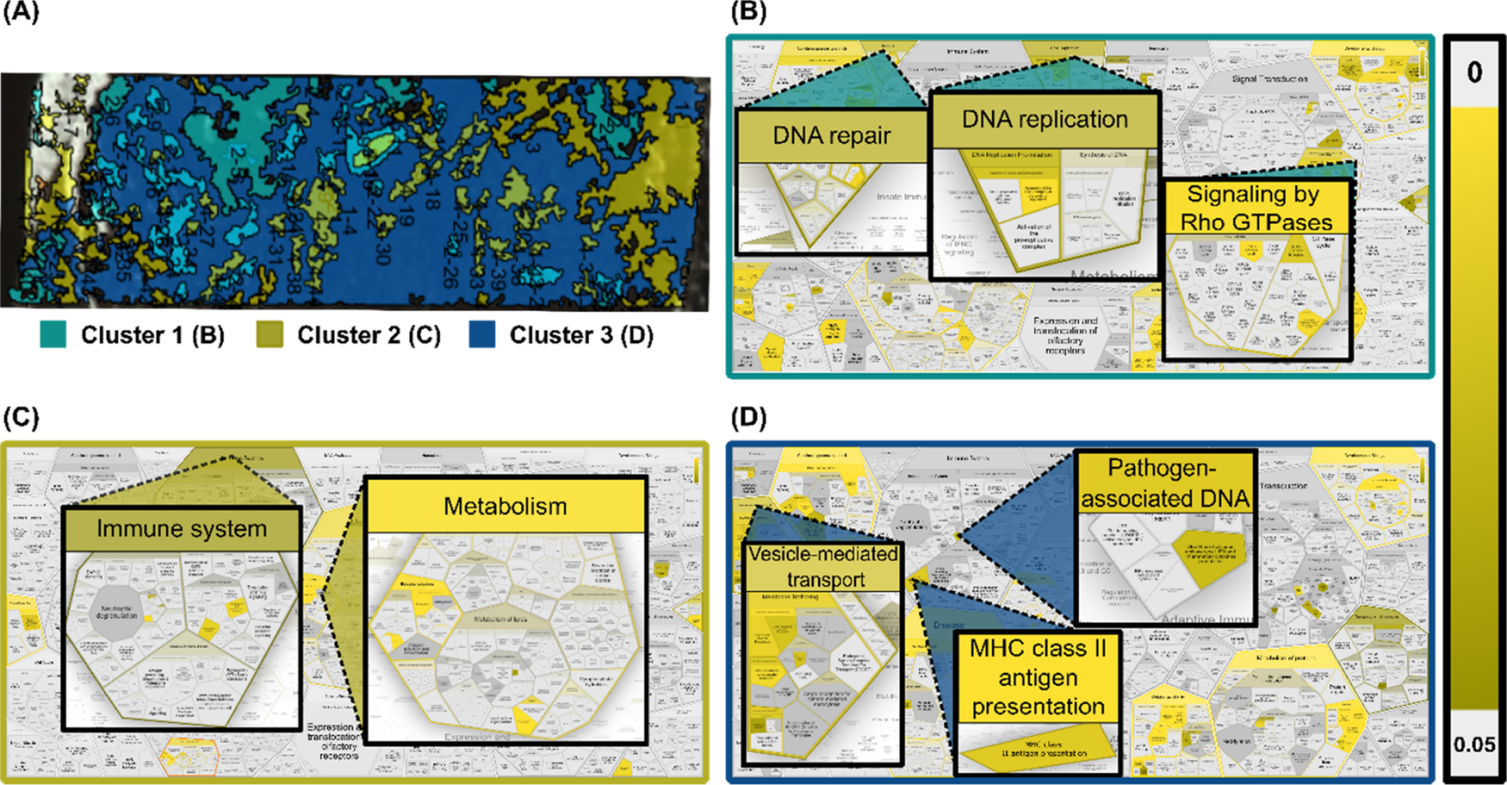
Cluster-specific pathway analysis of over-represented proteins from LMD LC-MS of 0.5 mm^2^ tissue of patient 1. (A) Overview of intensity clusters based on MALDI-IHC images. (B–D) Highlighted pathways correlating with over-represented proteins identified exclusively in cluster 1 to cluster 3, respectively. Scale bar = statistical significance of each hit pathway for the sample reflected in color gradient (*p*-value).

A great strength of the spatial omics approach used here is that the targeted multiplexed imaging of MALDI-IHC can be used to guide an untargeted bottom-up proteomics analysis of ROIs based on the localization of the PC-MTs. Here we investigated differentially regulated pathways that were specific for each of the three clusters identified based on the rapifleX measurements (Figure 5A). Based on the LC-MS identified proteins from each of the three clusters, IDs were extracted that were exclusive to a single cluster and pathway analysis was done using the Reactome knowledgebase tool for over-representation analysis (Figure 5B–D).^37^ Interestingly, we could identify a different pattern of over-represented pathways in each of the three clusters separately, some of which are highlighted in Figure 5.

Cluster 1, which corresponded mainly with PC-MTs for panCK, showed over-representation of proteins related to DNA repair and replication as well as signaling by Rho GTPases (Figure 5B). These pathways are of particular interest as the DNA repair and replication machinery has a large number of oncogenes and tumor suppressor genes, making them interesting in the context of cancer tissue. Rho GTPases are important modulators of cell signaling, including proliferation, survival, and death and are therefore also interesting targets in cancer biology.^38^ For cluster 2, which corresponded mainly with vimentin and CD20, there was an over-representation in proteins related to the immune system and general metabolism (Figure 5C). The correlation with immune system pathways is expected, as CD20 is a marker for B-cells and immune-related proteins will therefore be present. Metabolism-related pathways are often upregulated in cancer cells as they have a higher demand for energy compared to normal cells. Examples of this include the Warburg effect where aerobic glycolysis is increased and increased lipid biosynthesis.^39^ Finally, for cluster 3, corresponding mainly with actin-αSM, there was a correlation with pathways for vesicle-mediated transport, MHC class II antigen presentation and cytosolic sensors of pathogen-associated DNA. Vesicle-mediated transport, and particularly extracellular vesicles play an important role in cancer metastasis and tissue invasion through the formation of the actin-rich structures called invadopodia. These membrane protrusions help tumor cell invasion through the degradation of the extracellular matrix and could explain the over-representation of vesicle-mediated transport in this cluster.^40^ Under healthy condition, expression of MHC class II molecules is found only on antigen-presenting cells as part of the adaptive immune response by activation of T- or B-cells. However, MHC class II expression has also been recognized in certain cancer types, including breast cancer, and has been associated with improved progression-free survival and immune checkpoint inhibitor treatment.^41^ Regarding cytosolic sensors of pathogen-associated DNA, specifically DEx/H-box proteins are shown to be over-represented. These proteins are important for RNA metabolism and have been shown to activate type I interferons, resulting in inflammation. Furthermore, these proteins have also been implicated in cancer progression previously.^42^

Thus, the targeted imaging with MALDI-IHC complemented by an untargeted bottom-up proteomics analysis expands the amount of information gathered from a single tissue section. Here, the great variety in correlated pathways showed that localized tumor proteomics is important to fully understand the extent of intratumor heterogeneity and how it can drive disease progression.

## Conclusions

Here, we have developed and demonstrated a workflow for using targeted MALDI-IHC imaging to guide an untargeted bottom-up spatial proteomics analysis with LC-MS revealing extensive intratumor heterogeneity of over-represented pathways. The complementarity of the two approaches is promising with regard to the further development of the spatial omics field. Furthermore, untargeted imaging of on-tissue digested tissue revealed various peptides, which could be tentatively assigned to a wide range of proteins compared with our LC-MS data. Interestingly, vimentin, keratins, and actin could be tentatively identified based on multiple peptide fragments, which were also part of the antibody panel used for the targeted approach with the PC-MTs. No peptides were found related to HER2, CD20, or CD68 using the untargeted on-tissue digestion. The use of post-ionization with MALDI-2 showed an increased intensity of the PC-MTs up to a factor of two and showed potential for the development of new mass reporters. Although the on-tissue digestion covered a wider range of proteins, the MALDI-IHC allowed for easy detection and straightforward identification of proteins that were not detected in untargeted approaches. These panels could be customized and used for diagnostics or patient stratification with less chance of delocalization due to specific antibody binding. Combinations of multiplexed IHC with MALDI-IHC-guided proteomics could greatly expand our biological understanding of diseases by providing complementary information from the same tissue section without the need for customized instrumentation.

## Supporting Information Available

FFPE breast cancer sample information (OriGene) (Table S1); antibody panel information (Table S2); untargeted peptide MSI showed specific peptide distributions (Figure S1); single-pixel spectrum of the MALDI-IHC experiment on the rapifleX at 10 × 10 μm (Figure S2); multiplex MALDI-IHC on the rapifleX at 5 × 5 μm spatial resolution (Figure S3); comparison of HER2 fluorescent and MALDI-IHC images from the same tissue section (Figure S4); MALDI-IHC-guided LMD overview (Figure S5); and Venn diagram of the number of peaks detected with the different bottom-up proteomics approaches (Figure S6) (PDF)
