# Supplementary material for: MALDI-IHC-Guided In-Depth Spatial Proteomics: Targeted and Untargeted MSI Combined
Source: Anal Chem. 2023 Jan 13;95(4):2329–38. doi: 10.1021/acs.analchem.2c04220 (PMC9893213; doi:10.1021/acs.analchem.2c04220)
Supplement: Supplementary file 1 — ac2c04220_si_001.pdf [file ac2c04220_si_001.pdf]

Supplementary information:

MALDI-IHC guided in-depth spatial proteomics: targeted and untargeted MSI combined.

Britt S.R. Claes[1#], Kasper K. Krestensen[1#], Gargey Yagnik[2], Andrej Grgic[1], Christel Kuik[1], Mark J. Lim[2], Kenneth J. Rothschild[2,3], Michiel Vandenbosch[1], Ron M.A. Heeren[1]*

[1]The Maastricht MultiModal Molecular Imaging (M4I) institute, Division of Imaging Mass Spectrometry (IMS), Maastricht University, 6229 ER Maastricht, The Netherlands
[2]AmberGen, Inc., 44 Manning Road, Billerica, Massachusetts 01821, United States.
[3]Molecular Biophysics Laboratory, Department of Physics and Photonics Center, Boston University, Boston, Massachusetts 02215, United States

[#] These authors have equally contributed

* Corresponding author:
Prof. Ron M.A. Heeren
Email: r.heeren@maastrichtuniversity.nl

Table of Contents

Table S1: FFPE breast cancer sample information (OriGene). S2
Table S2: Antibody panel information S2
Figure S1: Untargeted peptide MSI showed specific peptide distributions S3
Figure S2: Single-pixel spectrum of the MALDI-IHC experiment on the Rapiflex at 10x10 µm S3
Figure S3: Multiplex MALDI-IHC on the rapifleX at 5x5 µm spatial resolution. S4
Figure S4: Comparison of HER2 fluorescent and MALDI-IHC images from the same tissue section S5
Figure S5: MALDI-IHC guided LMD overview S5
Figure S6: Venn diagram of the number of peaks detected with the different bottom-up approaches...... S6

Table S1: FFPE breast cancer sample information (OriGene).

Information	Patient 1 FR5B34765F	Patient 2 FR5B3391A2
PR status	+	-
ER status	+	-
HER2 status	-	+
HER2 strength	N.A.	Strong
Age	61	57
Gender	Female	Female
Sample type	FFPE block	FFPE block
Tissue origin/finding	Breast / Lymph node, axillary	Breast / Breast
Sample pathology from pathology verification	Adenocarcinoma of breast, ductal, lobular, metastatic	Adenocarcinoma of breast, ductal
Tumor grade	Nottingham G2: 6-7 points Intermediate combined grade (moderately favorable)	Nottingham G3: 8-9 points High combined grade (unfavorable)
TNM	T2N2aMX	pT2pN0pMX
Minimum stage grouping	IIIA	IIA
% Normal	5	3
% Lesion	0	0
% Tumor	95	85
% Tumor hypercellular stroma	0	0
% Tumor hypo/acellular stroma	0	2
% Necrosis	0	10
Race/Ethnicity	White or Caucasian	Not reported

Table S2: Antibody panel information used for the MALDI-IHC experiments.

Binding agent/ Fluorophore	Lot#	Quantity (conc.)	PC-MT (Da)	Binding Agent Vendor	Binding Agent Catalog	Species Reactivity
Actin-αSM / No Fluorophore	L2100055	12.5 µg (298 µg/mL)	1,194.66	ABCAM	ab220795	M, Rat, H
HER2 / DyLight 650	L2100034	12.5 µg (305 µg/mL)	1,210.74	ABCAM	ab251602	H
CD68 / No Fluorophore	L2100052	12.5 µg (400 µg/mL)	1,216.75	ABCAM	ab227458	H
Vimentin / No Fluorophore	L2100041	12.5 µg (316 µg/mL)	1,230.84	ABCAM	ab193555	M, Rat, H, AGM
PanCK / No Fluorophore	L2100043	12.5 µg (387 µg/mL)	1,638.87	ABCAM	ab264485	M, Rat, H
CD20 / No Fluorophore	L2100062	12.5 µg (260 µg/mL)	997.53	ABCAM	ab214282	H

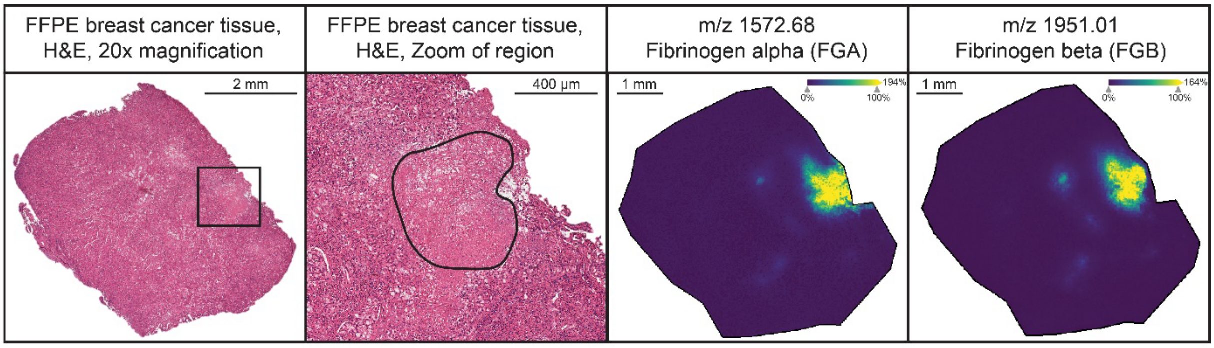

Figure S1: Untargeted peptide MSI showed specific peptide distributions within the necrotic area of the FFPE breast cancer tissue (based on the H&E, left) such as *m/z* 1572.68 and *m/z* 1951.01 (right), corresponding to fibrinogen alpha (FGA) and fibrinogen beta (FGB), respectively. The H&E was performed on a consecutive section.

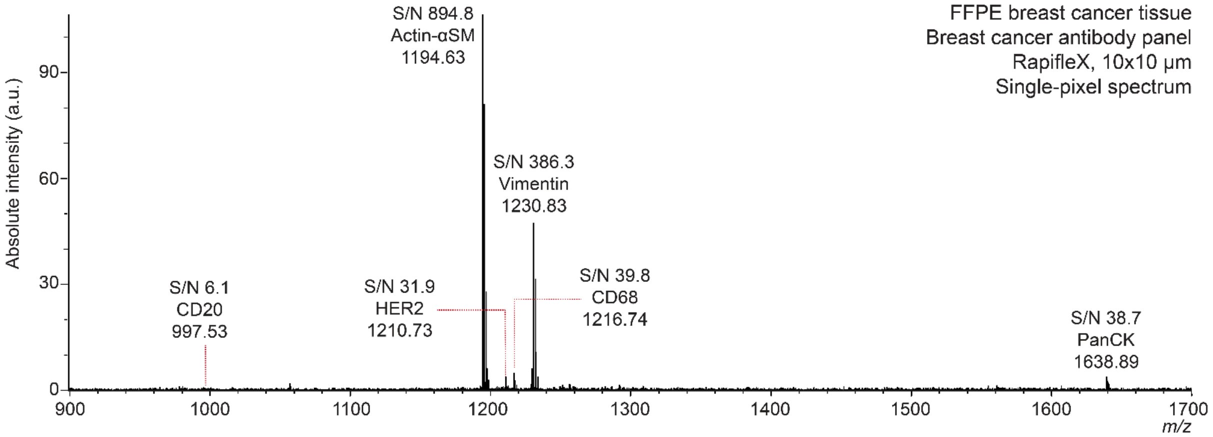

Figure S2: Single-pixel spectrum of the MALDI-IHC experiment on the rapifleX at 10x10 µm. The spectrum was TIC normalized in FlexImaging and exported to mMass, where peaks were picked and baseline corrected.

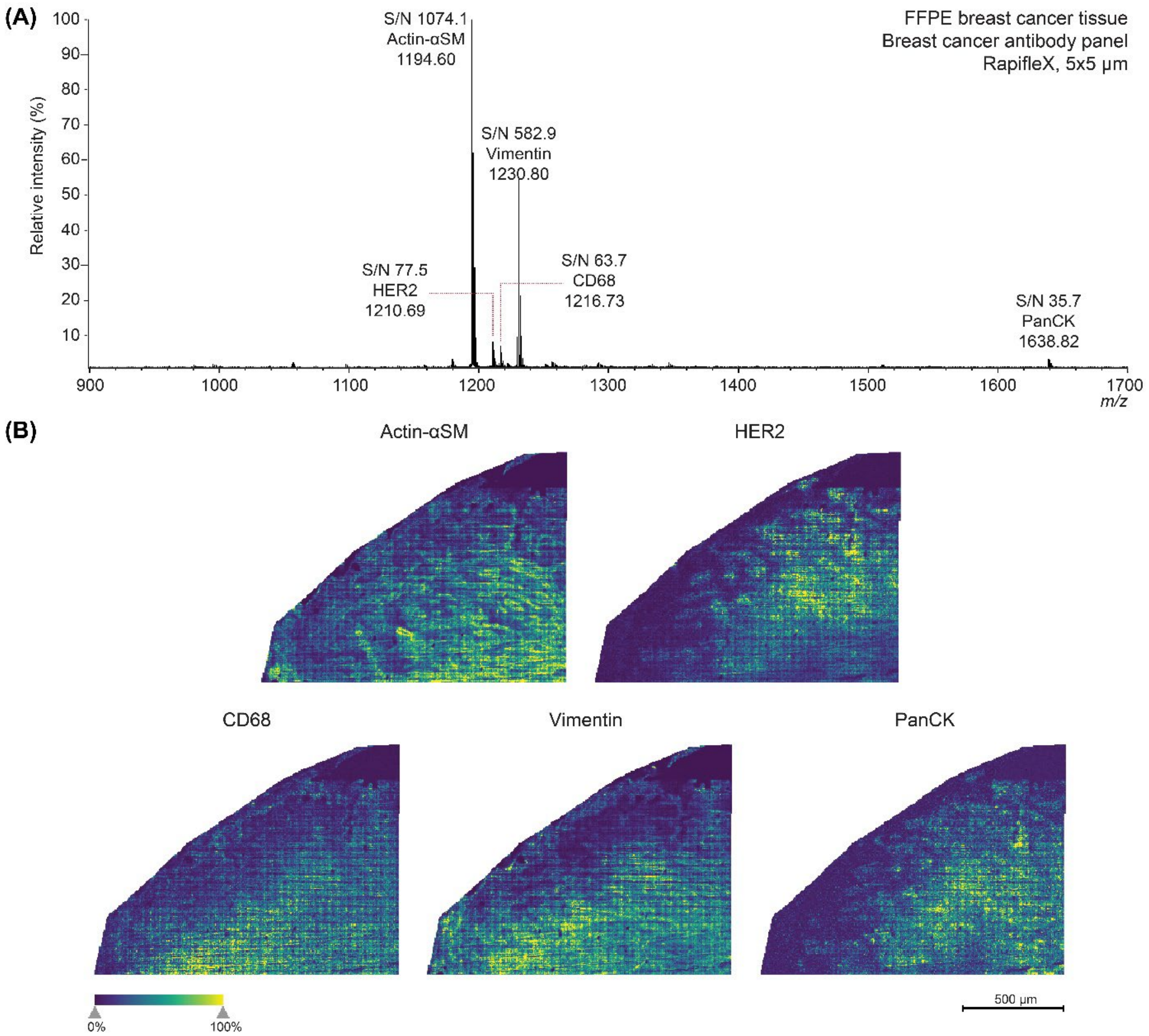

Figure S3: Multiplex MALDI-IHC on the rapifleX at 5x5 µm spatial resolution. (A) Spectrum of the MALDI-IHC measurement with the breast cancer antibody panel. Five out of six peptide mass reporters were detected. The spectrum was TIC normalized and baseline corrected. (B) Single-ion images of the five mass reporters were detected, which were actin-αSM, HER2, CD68, vimentin, and panCK, respectively. After optimization of the laser shots and frequency, oversampling could be avoided. However, a rastering pattern is still visible in the images due to the stage movement.

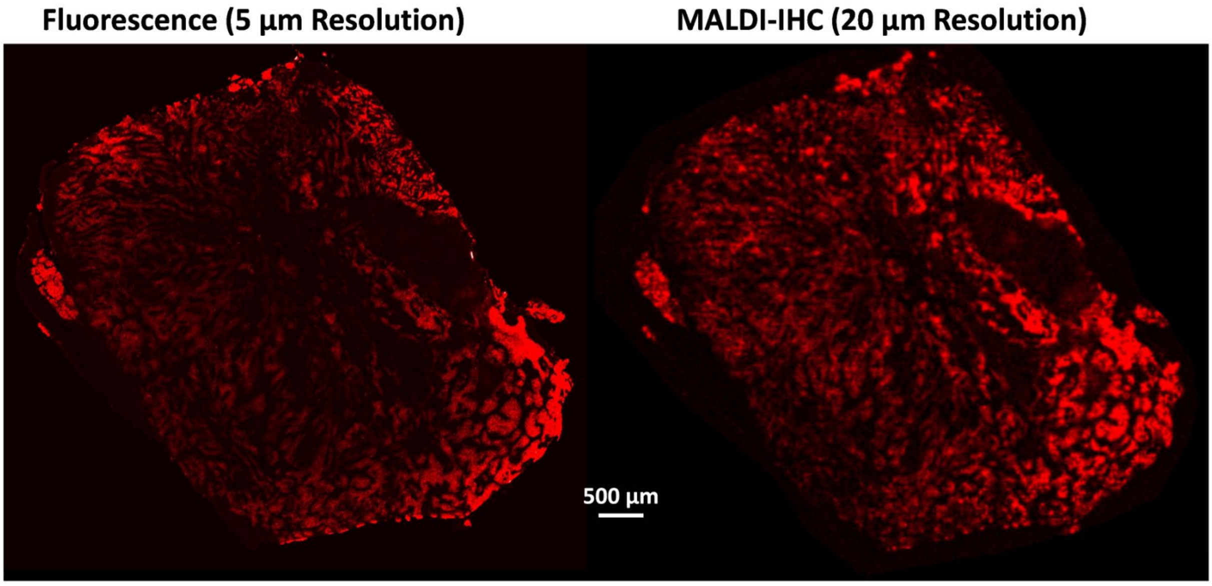

Figure S4: Comparison of HER2 fluorescent and MALDI-IHC images from the same tissue section stained using the six PC-MT antibody probes, of which the HER2 probe was dual-labeled with both a fluorophore and PC-MT (Table S2). The FFPE breast cancer tissue specimen was from Patient 2 (HER2+) and whole tissue section imaging was performed (note fluorescence imaging was performed at 5 µm resolution on a GenePix 4200A microarray scanner, Molecular Devices, San Jose, CA).

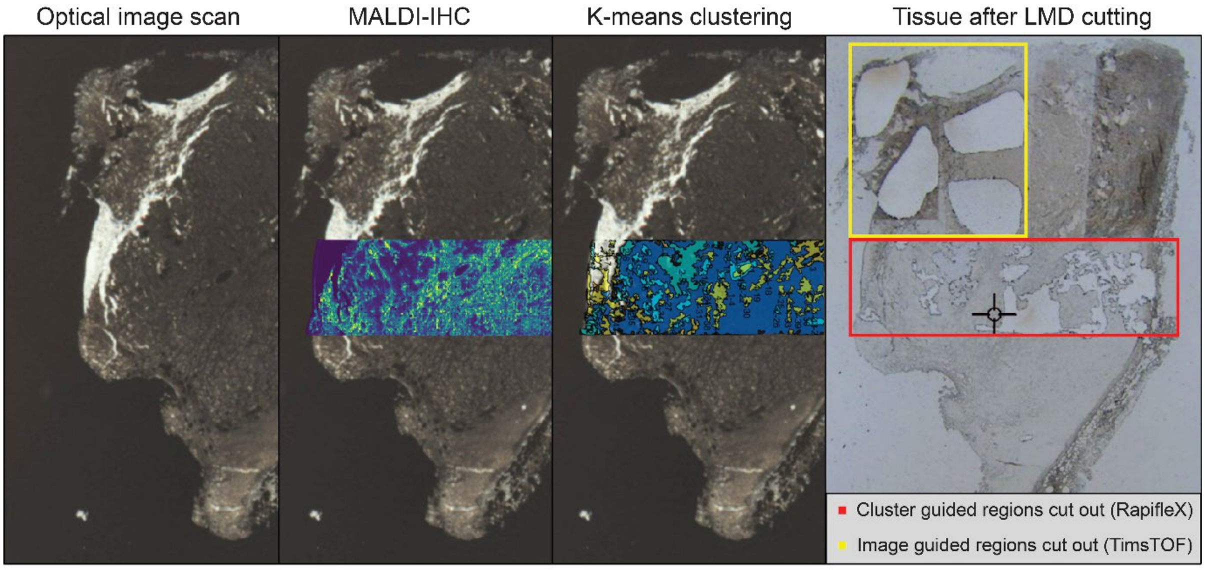

Figure S5: MALDI-IHC guided LMD overview. Visualization of each step from the optical image (panel 1) and MALDI-IHC (panel 2) to segmentation (panel 3) and laser-capture microdissection based on segmentation (panel 4).

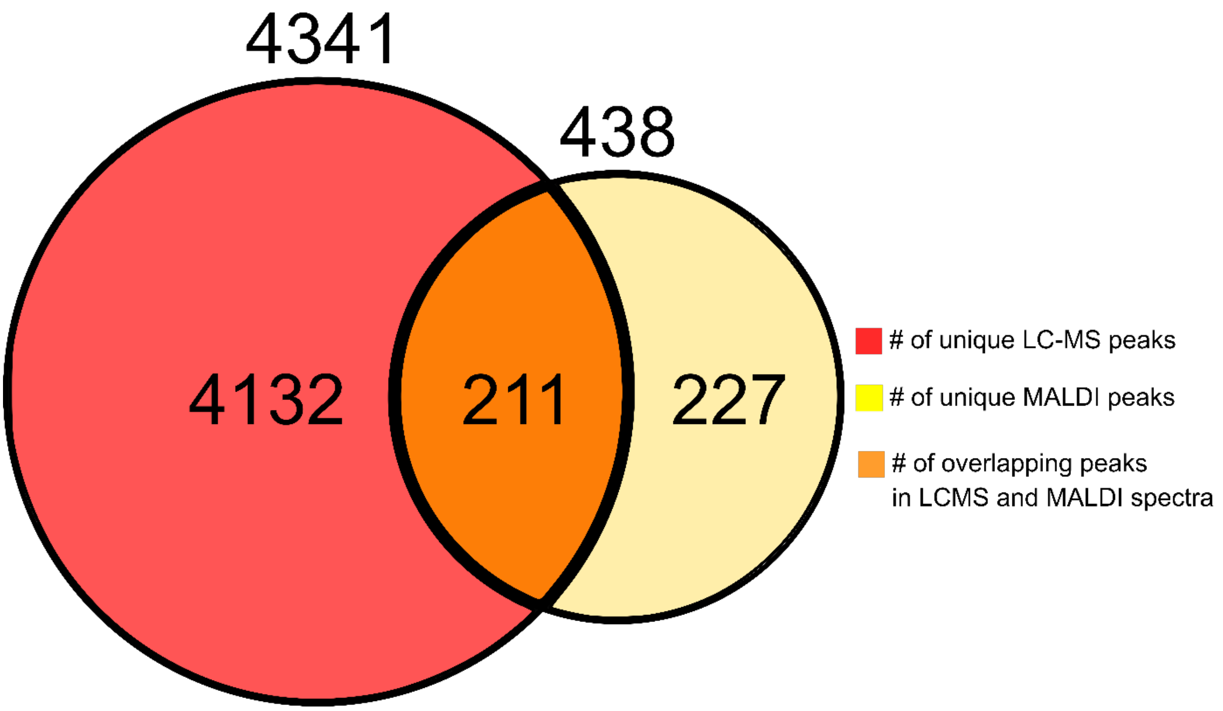

Figure S6: Venn diagram of the number of peaks detected with the different bottom-up approaches. With LMD followed by LC-MS, 4132 unique peaks were identified and correlated with peptides after data analysis, while the on-tissue digestion showed 227 unique peaks after peak picking. In total for both approaches, 4341 and 438 peaks were detected respectively, while comparing the two approaches, there was an overlap of 211 peptide masses, considering a +/- 20 ppm window.
